# 4-*tert*-Butyl­pyridinium picrate

**DOI:** 10.1107/S1600536810011037

**Published:** 2010-03-31

**Authors:** P. Ramesh, R. Akalya, A. Chandramohan, M. N. Ponnuswamy

**Affiliations:** a Centre of Advanced Study in Crystallography and Biophysics, University of Madras, Guindy Campus, Chennai 600 025, India; b Department of Chemistry, Sri Ramakrishna Mission Vidyalaya Arts and Science College, Coimbatore 641 020, India

## Abstract

In the title compound, C_9_H_14_N^+^·C_6_H_2_N_3_O_7_^−^, the three nitro groups of the picrate anion are twisted out of the plane of the attached benzene ring; the dihedral angles are 32.8 (2), 10.5 (4) and 12.3 (4)°. The pyridinium cations and picrate anions are linked *via* bifurcated N—H⋯(O,O) hydrogen bonds. The ionic pairs are linked into a ribbon-like structure along [101] by C—H⋯O hydrogen bonds.

## Related literature

For general background to picrate complexes, see: In *et al.* (1997 ▶); Zaderenko *et al.* (1997 ▶); Ashwell *et al.* (1995 ▶); Owen & White (1976 ▶); Shakir *et al.* (2009 ▶). 
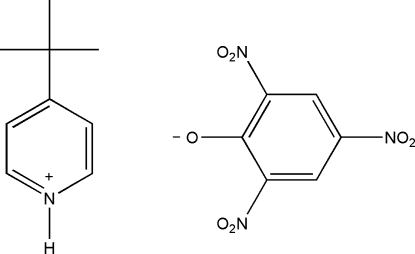


## Experimental

### 

#### Crystal data

C_9_H_14_N^+^·C_6_H_2_N_3_O_7_^−^
*M* *_r_* = 364.32
Monoclinic, 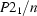

*a* = 5.7669 (12) Å
*b* = 26.798 (6) Å
*c* = 11.195 (3) Å
β = 97.335 (6)°
*V* = 1715.9 (7) Å^3^
*Z* = 4
Mo *K*α radiation
μ = 0.11 mm^−1^
*T* = 293 K
0.21 × 0.19 × 0.16 mm

#### Data collection

Bruker SMART APEXII area-detector diffractometer
Absorption correction: multi-scan (*SADABS*; Bruker, 2008 ▶) *T*_min_ = 0.976, *T*_max_ = 0.982
16875 measured reflections
4318 independent reflections
2677 reflections with *I* > 2σ(*I*)
*R*_int_ = 0.027

#### Refinement

*R*[*F* ^2^ > 2σ(*F* ^2^)] = 0.055
*wR*(*F* ^2^) = 0.178
*S* = 1.05
4318 reflections
242 parameters
H atoms treated by a mixture of independent and constrained refinement
Δρ_max_ = 0.48 e Å^−3^
Δρ_min_ = −0.18 e Å^−3^

### 

Data collection: *APEX2* (Bruker, 2008 ▶); cell refinement: *SAINT* (Bruker, 2008 ▶); data reduction: *SAINT*; program(s) used to solve structure: *SHELXS97* (Sheldrick, 2008 ▶); program(s) used to refine structure: *SHELXL97* (Sheldrick, 2008 ▶); molecular graphics: *ORTEP-3* (Farrugia, 1997 ▶); software used to prepare material for publication: *SHELXL97* and *PLATON* (Spek, 2009 ▶).

## Supplementary Material

Crystal structure: contains datablocks global, I. DOI: 10.1107/S1600536810011037/ci5056sup1.cif

Structure factors: contains datablocks I. DOI: 10.1107/S1600536810011037/ci5056Isup2.hkl

Additional supplementary materials:  crystallographic information; 3D view; checkCIF report

## Figures and Tables

**Table 1:** Hydrogen-bond geometry (Å, °)

*D*—H⋯*A*	*D*—H	H⋯*A*	*D*⋯*A*	*D*—H⋯*A*
N1—H1⋯O1^i^	0.91 (3)	1.85 (3)	2.659 (2)	148 (2)
N1—H1⋯O7^i^	0.91 (3)	2.38 (3)	3.085 (3)	135 (2)
C2—H2⋯O4^ii^	0.93	2.45	3.131 (3)	130
C6—H6⋯O1^iii^	0.93	2.42	3.137 (3)	133

Symmetry codes: (i) 
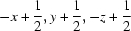
; (ii) 
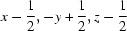
; (iii) 
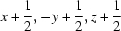
.

**Fig. 1.**
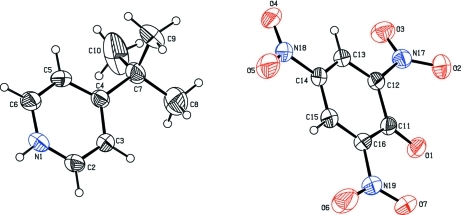
The asymmetric unit of the title compound. Displacement ellipsoids are drawn at the 50% probability level.

**Fig. 2.**
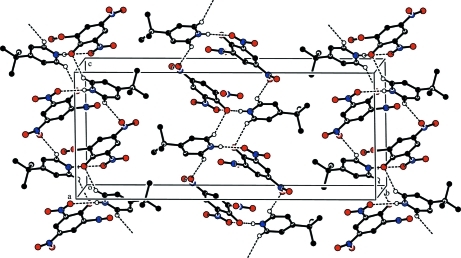
The crystal packing of the title compound, viewed down the a axis. H atoms not involved in hydrogen bonding (dashed lines) have been omitted for clarity.

## supplementary crystallographic information

### Comment

It is well known that picric acid forms charge transfer molecular complexes with a number of aromatic compounds such as aromatic hydrocarbons and amines, through electrostatic or hydrogen bonding interactions (In *et al.*, 1997; Zaderenko *et al.*, 1997). The bonding of donor-acceptor picric acid complexes strongly depends on the nature of partners. Some of the picric acid complexes crystallize in centrosymmetric space group owing to non-linear optical properties (NLO) (Shakir *et al.*, 2009). This is due to the aggregation of the donor-acceptor molecules in a non-centrosymmetric manner which contributes to the bulk susceptibility from intermolecular charge transfer (Ashwell *et al.*,1995; Owen & White, 1976). We report here the crystal structure of the title salt.

The pyridinium ring of the cation (Fig.1) is planar (r.m.s. deviation 0.019 Å). In the picrate anion, the keto O atom deviates from the benzene plane by 0.139 (3) Å. The C11—O1 bond [1.245 (2) Å] assumes a partial double bond character. The C11—C12 [1.452 (3) Å] and C11—C16 [1.436 (3) Å] bond distances are longer than the other C—C bond lengths of the benzene ring. The three nitro groups are twisted out of the attached benzene ring by 32.8 (2)° [N17/O2/O3], 10.5 (4)° [N18/O4/O5] and 12.3 (4)° [N19/O6/O7].

In the crystal, the cations and anions are linked via N—H···O hydrogen bonds involving the phenolate O atom and one of the nitro O atoms. The ionic pairs are linked into a ribbon-like structure along the [101] by C—H···O hydrogen bonds (Fig.2).

### Experimental

Equimolar solutions of 4-tertiarybutyl pyridine in methanol and picric acid in methanol were mixed together and the solution was stirred well for 1 h and the precipitated salt was filtered off. The salt was repeatedly recrystallised from methanol to get single crystals suitable for X-ray analysis.

### Refinement

The N-bound H atom was located in a difference map and refined freely. C-bound H atoms were positioned geometrically (C–H = 0.93-0.96 Å) and allowed to ride on their parent atoms, with U_iso_(H) = 1.5U_eq_(C) for methyl H and 1.2U_eq_(C) for other H atoms. The displacement ellipsoids for the methyl carbons (C8-C10) of the tert-butyl group are elongated, suggesting possible disorder i.e free rotation of the tert-butyl group. Attempts to model the tert-butyl group as disordered over two sites did not give satisfactory results. Hence the original model was retained.

### Crystal data

**Table:**

C_9_H_14_N^+^·C_6_H_2_N_3_O_7_^−^	*F*(000) = 760
*M**_r_* = 364.32	*D*_x_ = 1.410 Mg m^−^^3^
Monoclinic, *P*2_1_/*n*	Mo *K*α radiation, λ = 0.71073 Å
Hall symbol: -P 2yn	Cell parameters from 2565 reflections
*a* = 5.7669 (12) Å	θ = 1.5–28.5°
*b* = 26.798 (6) Å	µ = 0.11 mm^−^^1^
*c* = 11.195 (3) Å	*T* = 293 K
β = 97.335 (6)°	Block, colourless
*V* = 1715.9 (7) Å^3^	0.21 × 0.19 × 0.16 mm
*Z* = 4	

### Data collection

**Table:**

Bruker SMART APEXII area-detector diffractometer	4318 independent reflections
Radiation source: fine-focus sealed tube	2677 reflections with *I* > 2σ(*I*)
graphite	*R*_int_ = 0.027
ω and φ scans	θ_max_ = 28.5°, θ_min_ = 1.5°
Absorption correction: multi-scan (*SADABS*; Bruker, 2008)	*h* = −7→7
*T*_min_ = 0.976, *T*_max_ = 0.982	*k* = −34→35
16875 measured reflections	*l* = −14→15

### Refinement

**Table:**

Refinement on *F*^2^	Primary atom site location: structure-invariant direct methods
Least-squares matrix: full	Secondary atom site location: difference Fourier map
*R*[*F*^2^ > 2σ(*F*^2^)] = 0.055	Hydrogen site location: inferred from neighbouring sites
*wR*(*F*^2^) = 0.178	H atoms treated by a mixture of independent and constrained refinement
*S* = 1.05	*w* = 1/[σ^2^(*F*_o_^2^) + (0.0841*P*)^2^ + 0.3243*P*] where *P* = (*F*_o_^2^ + 2*F*_c_^2^)/3
4318 reflections	(Δ/σ)_max_ = 0.001
242 parameters	Δρ_max_ = 0.48 e Å^−^^3^
0 restraints	Δρ_min_ = −0.18 e Å^−^^3^

### Special details

**Table:**

Geometry. All esds (except the esd in the dihedral angle between two l.s. planes) are estimated using the full covariance matrix. The cell esds are taken into account individually in the estimation of esds in distances, angles and torsion angles; correlations between esds in cell parameters are only used when they are defined by crystal symmetry. An approximate (isotropic) treatment of cell esds is used for estimating esds involving l.s. planes.
Refinement. Refinement of *F*^2^ against ALL reflections. The weighted *R*-factor wR and goodness of fit *S* are based on *F*^2^, conventional *R*-factors *R* are based on *F*, with *F* set to zero for negative *F*^2^. The threshold expression of *F*^2^ > σ(*F*^2^) is used only for calculating *R*-factors(gt) etc. and is not relevant to the choice of reflections for refinement. *R*-factors based on *F*^2^ are statistically about twice as large as those based on *F*, and *R*- factors based on ALL data will be even larger.

### Fractional atomic coordinates and isotropic or equivalent isotropic displacement parameters (Å^2^)

**Table:**

	*x*	*y*	*z*	*U*_iso_*/*U*_eq_	
O1	0.2606 (3)	0.00115 (5)	0.17613 (13)	0.0663 (4)	
O2	0.5627 (3)	−0.05436 (6)	0.32430 (15)	0.0744 (4)	
O3	0.8672 (3)	−0.00990 (7)	0.37736 (16)	0.0800 (5)	
O4	0.7078 (4)	0.14493 (6)	0.57283 (18)	0.0991 (7)	
O5	0.3706 (4)	0.17951 (7)	0.5339 (2)	0.1081 (7)	
O6	−0.1374 (4)	0.12612 (8)	0.1958 (3)	0.1319 (10)	
O7	−0.1413 (3)	0.05113 (7)	0.13505 (15)	0.0829 (5)	
N1	0.5892 (3)	0.43662 (7)	0.37124 (16)	0.0627 (4)	
H1	0.516 (5)	0.4666 (11)	0.362 (3)	0.097 (9)*	
C2	0.5004 (4)	0.39966 (9)	0.3030 (2)	0.0669 (6)	
H2	0.3705	0.4053	0.2463	0.080*	
C3	0.5967 (4)	0.35326 (8)	0.31436 (19)	0.0631 (5)	
H3	0.5356	0.3277	0.2637	0.076*	
C4	0.7843 (3)	0.34401 (7)	0.40070 (18)	0.0545 (5)	
C5	0.8766 (4)	0.38456 (8)	0.46659 (19)	0.0667 (6)	
H5	1.0077	0.3803	0.5233	0.080*	
C6	0.7790 (4)	0.43054 (9)	0.45000 (19)	0.0715 (6)	
H6	0.8451	0.4577	0.4937	0.086*	
C7	0.8842 (4)	0.29206 (8)	0.4249 (2)	0.0732 (6)	
C8	0.7573 (6)	0.25297 (11)	0.3428 (4)	0.1142 (11)	
H8A	0.7858	0.2589	0.2613	0.171*	
H8B	0.5925	0.2549	0.3475	0.171*	
H8C	0.8134	0.2204	0.3677	0.171*	
C9	0.8433 (7)	0.27804 (12)	0.5529 (3)	0.1227 (12)	
H9A	0.8943	0.2444	0.5696	0.184*	
H9B	0.6797	0.2807	0.5603	0.184*	
H9C	0.9302	0.3002	0.6092	0.184*	
C10	1.1361 (5)	0.29230 (13)	0.4139 (6)	0.179 (3)	
H10A	1.1620	0.3094	0.3415	0.268*	
H10B	1.1913	0.2586	0.4110	0.268*	
H10C	1.2193	0.3090	0.4822	0.268*	
C11	0.3052 (3)	0.03289 (7)	0.25697 (16)	0.0529 (4)	
C12	0.5135 (3)	0.03034 (7)	0.34450 (16)	0.0508 (4)	
C13	0.5836 (3)	0.06717 (7)	0.42427 (17)	0.0545 (5)	
H13	0.7245	0.0645	0.4745	0.065*	
C14	0.4436 (4)	0.10853 (7)	0.42986 (18)	0.0594 (5)	
C15	0.2372 (4)	0.11350 (7)	0.35493 (19)	0.0615 (5)	
H15	0.1429	0.1413	0.3608	0.074*	
C16	0.1713 (3)	0.07717 (7)	0.27149 (17)	0.0568 (5)	
N17	0.6573 (3)	−0.01424 (6)	0.34792 (14)	0.0580 (4)	
N18	0.5107 (4)	0.14682 (7)	0.51824 (18)	0.0770 (6)	
N19	−0.0483 (3)	0.08495 (8)	0.19498 (17)	0.0707 (5)	

### Atomic displacement parameters (Å^2^)

**Table:**

	*U*^11^	*U*^22^	*U*^33^	*U*^12^	*U*^13^	*U*^23^
O1	0.0751 (9)	0.0638 (9)	0.0581 (8)	−0.0153 (7)	0.0019 (7)	−0.0106 (7)
O2	0.0854 (10)	0.0527 (9)	0.0874 (11)	−0.0013 (7)	0.0194 (8)	−0.0060 (8)
O3	0.0656 (10)	0.0859 (12)	0.0845 (11)	0.0082 (8)	−0.0058 (8)	−0.0085 (9)
O4	0.1186 (14)	0.0598 (10)	0.1032 (13)	−0.0103 (9)	−0.0463 (12)	−0.0089 (9)
O5	0.1340 (17)	0.0605 (11)	0.1192 (16)	0.0156 (11)	−0.0245 (13)	−0.0300 (10)
O6	0.1079 (15)	0.0853 (15)	0.181 (2)	0.0249 (12)	−0.0669 (16)	−0.0217 (14)
O7	0.0782 (10)	0.0846 (11)	0.0774 (10)	−0.0123 (9)	−0.0226 (8)	−0.0048 (9)
N1	0.0751 (11)	0.0559 (10)	0.0575 (9)	0.0129 (9)	0.0101 (8)	0.0106 (8)
C2	0.0577 (11)	0.0668 (13)	0.0720 (13)	0.0010 (10)	−0.0075 (9)	0.0177 (11)
C3	0.0625 (12)	0.0542 (12)	0.0683 (12)	−0.0073 (9)	−0.0087 (9)	0.0063 (9)
C4	0.0507 (10)	0.0532 (10)	0.0587 (11)	0.0022 (8)	0.0038 (8)	0.0099 (8)
C5	0.0692 (13)	0.0670 (13)	0.0579 (11)	0.0038 (10)	−0.0148 (9)	0.0055 (10)
C6	0.0972 (16)	0.0614 (13)	0.0527 (11)	0.0015 (11)	−0.0024 (11)	−0.0025 (9)
C7	0.0648 (12)	0.0551 (12)	0.0963 (17)	0.0080 (10)	−0.0022 (11)	0.0165 (11)
C8	0.122 (3)	0.0661 (17)	0.150 (3)	0.0173 (16)	0.000 (2)	−0.0063 (18)
C9	0.161 (3)	0.084 (2)	0.114 (2)	0.001 (2)	−0.014 (2)	0.0452 (18)
C10	0.0698 (19)	0.087 (2)	0.386 (8)	0.0310 (16)	0.058 (3)	0.059 (3)
C11	0.0595 (11)	0.0507 (10)	0.0481 (9)	−0.0149 (8)	0.0051 (8)	0.0020 (8)
C12	0.0559 (10)	0.0482 (10)	0.0483 (9)	−0.0056 (8)	0.0069 (8)	0.0042 (8)
C13	0.0596 (10)	0.0497 (10)	0.0516 (10)	−0.0089 (8)	−0.0024 (8)	0.0057 (8)
C14	0.0733 (12)	0.0422 (10)	0.0590 (11)	−0.0101 (9)	−0.0054 (9)	0.0005 (8)
C15	0.0681 (12)	0.0457 (10)	0.0678 (12)	−0.0029 (9)	−0.0026 (9)	0.0033 (9)
C16	0.0572 (10)	0.0544 (11)	0.0560 (10)	−0.0081 (9)	−0.0042 (8)	0.0057 (9)
N17	0.0661 (11)	0.0581 (10)	0.0504 (9)	−0.0002 (8)	0.0096 (7)	−0.0010 (7)
N18	0.1021 (15)	0.0435 (10)	0.0770 (12)	−0.0075 (9)	−0.0203 (11)	−0.0017 (8)
N19	0.0655 (11)	0.0675 (12)	0.0741 (12)	−0.0054 (9)	−0.0102 (9)	0.0049 (9)

### Geometric parameters (Å, °)

**Table:**

O1—C11	1.245 (2)	C7—C9	1.529 (4)
O2—N17	1.219 (2)	C8—H8A	0.96
O3—N17	1.219 (2)	C8—H8B	0.96
O4—N18	1.221 (3)	C8—H8C	0.96
O5—N18	1.219 (3)	C9—H9A	0.96
O6—N19	1.218 (3)	C9—H9B	0.96
O7—N19	1.211 (2)	C9—H9C	0.96
N1—C2	1.314 (3)	C10—H10A	0.96
N1—C6	1.325 (3)	C10—H10B	0.96
N1—H1	0.91 (3)	C10—H10C	0.96
C2—C3	1.361 (3)	C11—C16	1.436 (3)
C2—H2	0.93	C11—C12	1.452 (3)
C3—C4	1.378 (3)	C12—C13	1.357 (3)
C3—H3	0.93	C12—N17	1.452 (3)
C4—C5	1.381 (3)	C13—C14	1.377 (3)
C4—C7	1.518 (3)	C13—H13	0.93
C5—C6	1.357 (3)	C14—C15	1.372 (3)
C5—H5	0.93	C14—N18	1.444 (3)
C6—H6	0.93	C15—C16	1.369 (3)
C7—C10	1.473 (4)	C15—H15	0.93
C7—C8	1.519 (4)	C16—N19	1.451 (3)
			
C2—N1—C6	121.45 (19)	H9B—C9—H9C	109.5
C2—N1—H1	117.2 (18)	C7—C10—H10A	109.5
C6—N1—H1	121.3 (18)	C7—C10—H10B	109.5
N1—C2—C3	120.67 (18)	H10A—C10—H10B	109.5
N1—C2—H2	119.7	C7—C10—H10C	109.5
C3—C2—H2	119.7	H10A—C10—H10C	109.5
C2—C3—C4	120.3 (2)	H10B—C10—H10C	109.5
C2—C3—H3	119.9	O1—C11—C16	125.64 (17)
C4—C3—H3	119.9	O1—C11—C12	122.35 (18)
C3—C4—C5	116.58 (18)	C16—C11—C12	111.90 (16)
C3—C4—C7	122.42 (19)	C13—C12—C11	124.14 (17)
C5—C4—C7	120.99 (18)	C13—C12—N17	117.36 (16)
C6—C5—C4	121.09 (18)	C11—C12—N17	118.50 (16)
C6—C5—H5	119.5	C12—C13—C14	119.20 (17)
C4—C5—H5	119.5	C12—C13—H13	120.4
N1—C6—C5	119.7 (2)	C14—C13—H13	120.4
N1—C6—H6	120.2	C15—C14—C13	121.19 (18)
C5—C6—H6	120.2	C15—C14—N18	119.05 (19)
C10—C7—C4	109.7 (2)	C13—C14—N18	119.73 (18)
C10—C7—C8	110.9 (3)	C16—C15—C14	119.45 (19)
C4—C7—C8	112.5 (2)	C16—C15—H15	120.3
C10—C7—C9	110.5 (3)	C14—C15—H15	120.3
C4—C7—C9	107.0 (2)	C15—C16—C11	123.94 (17)
C8—C7—C9	106.1 (2)	C15—C16—N19	116.49 (18)
C7—C8—H8A	109.5	C11—C16—N19	119.57 (17)
C7—C8—H8B	109.5	O3—N17—O2	122.98 (18)
H8A—C8—H8B	109.5	O3—N17—C12	118.23 (17)
C7—C8—H8C	109.5	O2—N17—C12	118.77 (17)
H8A—C8—H8C	109.5	O5—N18—O4	123.3 (2)
H8B—C8—H8C	109.5	O5—N18—C14	119.00 (19)
C7—C9—H9A	109.5	O4—N18—C14	117.7 (2)
C7—C9—H9B	109.5	O7—N19—O6	121.66 (19)
H9A—C9—H9B	109.5	O7—N19—C16	120.72 (19)
C7—C9—H9C	109.5	O6—N19—C16	117.60 (19)
H9A—C9—H9C	109.5		
			
C6—N1—C2—C3	2.4 (3)	C12—C13—C14—N18	177.02 (19)
N1—C2—C3—C4	2.2 (3)	C13—C14—C15—C16	−1.3 (3)
C2—C3—C4—C5	−4.7 (3)	N18—C14—C15—C16	−179.48 (19)
C2—C3—C4—C7	174.3 (2)	C14—C15—C16—C11	0.5 (3)
C3—C4—C5—C6	2.9 (3)	C14—C15—C16—N19	−179.75 (19)
C7—C4—C5—C6	−176.1 (2)	O1—C11—C16—C15	−173.87 (19)
C2—N1—C6—C5	−4.2 (3)	C12—C11—C16—C15	2.3 (3)
C4—C5—C6—N1	1.5 (4)	O1—C11—C16—N19	6.4 (3)
C3—C4—C7—C10	124.9 (3)	C12—C11—C16—N19	−177.37 (16)
C5—C4—C7—C10	−56.2 (4)	C13—C12—N17—O3	−31.6 (2)
C3—C4—C7—C8	0.9 (3)	C11—C12—N17—O3	148.47 (18)
C5—C4—C7—C8	179.9 (2)	C13—C12—N17—O2	146.61 (18)
C3—C4—C7—C9	−115.2 (3)	C11—C12—N17—O2	−33.3 (2)
C5—C4—C7—C9	63.7 (3)	C15—C14—N18—O5	8.6 (3)
O1—C11—C12—C13	171.42 (18)	C13—C14—N18—O5	−169.7 (2)
C16—C11—C12—C13	−4.9 (3)	C15—C14—N18—O4	−170.6 (2)
O1—C11—C12—N17	−8.6 (3)	C13—C14—N18—O4	11.1 (3)
C16—C11—C12—N17	175.02 (16)	C15—C16—N19—O7	−166.1 (2)
C11—C12—C13—C14	4.5 (3)	C11—C16—N19—O7	13.6 (3)
N17—C12—C13—C14	−175.42 (17)	C15—C16—N19—O6	12.1 (3)
C12—C13—C14—C15	−1.2 (3)	C11—C16—N19—O6	−168.2 (2)

### Hydrogen-bond geometry (Å, °)

**Table:**

*D*—H···*A*	*D*—H	H···*A*	*D*···*A*	*D*—H···*A*
N1—H1···O1^i^	0.91 (3)	1.85 (3)	2.659 (2)	148 (2)
N1—H1···O7^i^	0.91 (3)	2.38 (3)	3.085 (3)	135 (2)
C2—H2···O4^ii^	0.93	2.45	3.131 (3)	130
C6—H6···O1^iii^	0.93	2.42	3.137 (3)	133

Symmetry codes: (i) −*x*+1/2, *y*+1/2, −*z*+1/2; (ii) *x*−1/2, −*y*+1/2, *z*−1/2; (iii) *x*+1/2, −*y*+1/2, *z*+1/2.
